# Assessing the Integrity of Clinical Trials Included in Evidence Syntheses

**DOI:** 10.3390/ijerph20126138

**Published:** 2023-06-15

**Authors:** María Núñez-Núñez, Naomi Cano-Ibáñez, Javier Zamora, Aurora Bueno-Cavanillas, Khalid Saeed Khan

**Affiliations:** 1 Pharmacy Department, Clínico San Cecilio University Hospital, 18016 Granada, Spain; 2 Biosanitary Research Institute (Ibs. Granada), 18012 Granada, Spain; 3 Consortium for Biomedical Research in Epidemiology and Public Health (CIBERESP), 28029 Madrid, Spain; 4 Department of Preventive Medicine and Public Health, University of Granada, 18016 Granada, Spain; 5 Department of Biostatistics, Ramón y Cajal University Hospital (IRYCIS), 28034 Madrid, Spain; 6 Institute of Metabolism and Systems Research, University of Birmingham, Birmingham B15 2TT, UK

**Keywords:** research integrity, evidence synthesis, systematic reviews, clinical trials

## Abstract

Evidence syntheses of randomized clinical trials (RCTs) offer the highest level of scientific evidence for informing clinical practice and policy. The value of evidence synthesis itself depends on the trustworthiness of the included RCTs. The rising number of retractions and expressions of concern about the authenticity of RCTs has raised awareness about the existence of problematic studies, sometimes called “zombie” trials. Research integrity, i.e., adherence to ethical and professional standards, is a multi-dimensional concept that is incompletely evaluated for the RCTs included in current evidence syntheses. Systematic reviewers tend to rely on the editorial and peer-review system established by journals as custodians of integrity of the RCTs they synthesize. It is now well established that falsified and fabricated RCTs are slipping through. Thus, RCT integrity assessment becomes a necessary step in systematic reviews going forward, in particular because RCTs with data-related integrity concerns remain available for use in evidence syntheses. There is a need for validated tools for systematic reviewers to proactively deploy in the assessment of integrity deviations without having to wait for RCTs to be retracted by journals or expressions of concern issued. This article analyzes the issues and challenges in conducting evidence syntheses where the literature contains RCTs with possible integrity deficits. The way forward in the form of formal RCT integrity assessments in systematic reviews is proposed, and implications of this new initiative are discussed. Future directions include emphasizing ethical and professional standards, providing tailored integrity-specific training, and creating systems to promote research integrity, as improvements in RCT integrity will benefit evidence syntheses.

## 1. Introduction

Evidence syntheses of randomized clinical trials (RCTs) offer the highest level of effectiveness evidence validity for informing clinical practice and policy [1]. They make the results of RCTs available to practitioners and allow them to reach patients through systematic reviews and clinical practice guidelines. The rising number of allegations of data fabrication and falsification in retractions and expressions of concern about questionable research practices or faulty research methodology [2,3,4,5] has raised awareness about the authenticity of RCTs. (Un)intentional errors may lead to the existence of problematic studies, sometimes called “zombie” trials [6], within the literature. It has recently been recognized that: “*Even though the process for the detection and correction of error and fraud might be fairly well established and “standardized”, such as in COPE or ICMJE guidelines, inter-journal and inter-publisher variability, including editorial responsibilities, will continue to limit the effective correction of erroneous and fraudulent literature globally*” [7]. This background has important implications for evidence syntheses, including both systematic reviews and guidelines, that have the potential to widen the impact of faulty RCTs. This commentary describes challenges in evidence syntheses, with respect to included RCT integrity, with particular focus on data fabrication and falsification.

## 2. How Can We Trust Evidence Syntheses?

**Figure 1:**
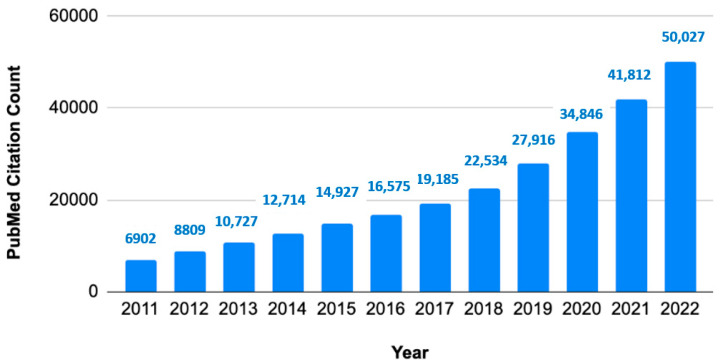
Growth of systematic reviews to synthesize evidence. Annual citation counts of article type systematic reviews in PubMed database.

Evidence synthesis is a type of research method that collates all relevant studies and interprets their collective findings. There has been sustained growth in systematic review publications during the last decade (Figure 1).

The fundamental steps of evidence synthesis are as follows: defining the question(s); searching for relevant studies (screening and selection against defined inclusion and exclusion criteria); appraising the quality of the studies included and extracting relevant data; collating data, undertaking meta-analyses where appropriate; and interpreting the findings [1]. The approach to quality assessment of the evidence attempts to minimize the risk of bias through the use of an explicit and transparent methodology. A typical example of evidence synthesis is a systematic review of treatment effectiveness [8]. The product will be a guideline which provides evidence-based statements for clinical decision-making, practice, and policy. The critical appraisal of evidence is key to the derivation of trustworthy practice recommendations and is the essence of evidence-based medicine [9].

The evidence used in the systematic reviews may include a range of study designs, from RCTs to observational studies, including case series too. RCTs are ranked the highest in the hierarchy of evidential validity due to their unique design that randomly assigns participants into experimental or control groups to compare outcomes. Randomization targets the minimization of selection bias in generating evidence about the effectiveness of interventions. To robustly implement and report trials, researchers performing RCTs are required to undertake regular training (good clinical practice courses), and trials are required to be prospectively registered in registries such as Clinicaltrials.gov, amongst other requirements [10,11]. Therefore, whenever available, systematic reviews and guidelines endeavor to include RCTs over other designs [12,13]. To enable the appraisal of evidence syntheses, several instruments or tools exist. As examples of tools for assessing systematic reviews, two widely used tools are AMSTAR-2 and ROBIS [14,15]. Other examples of tools for practicing guidelines are RIGHT and AGREE [16,17]. These tools include domains on how studies included in the synthesis were identified, selected, appraised, and analyzed, among others. They cover the risk of bias assessment. Nevertheless, the risk of bias assessment only partially targets integrity as the two concepts are not synonymous. They do not explicitly target study integrity assessment within evidence syntheses, a topic on which this paper will focus.

## 3. Relationship between Primary Research Integrity and Evidence Syntheses

Research integrity is a term that captures compliance with ethical and professional standards in the conduct of scientific studies [18]. In order to elucidate the research integrity concept, we provide, in Table 1, key research integrity terms and definitions.

Focusing evidence syntheses on the best available study designs, carrying out selected study appraisal, and basing inferences on the highest quality subgroup of studies targets avoidance or minimization of the risk of bias. Current review methodology does not explicitly target study integrity. It is important to recognize that, conceptually, study quality assessment and integrity evaluation are not synonymous. Inherently, systematic reviewers tend to rely on the editorial and peer-review systems established by journals, which appear as a custodian of research integrity assessment. However, there are well-known gaps in journals’ authors’ instructions, editorial and peer-review evaluations, and investigation policies about post-publication allegations of scientific misconduct [22]. Study integrity assessment can be time-consuming, permitting fabricated or falsified studies to remain usable and to be included into evidence syntheses [23]. Only 5% of systematic reviews or clinical practice guidelines have corrected or retracted their results, with respect to retractions of included studies [24]. Without actual integrity assessment, the underpinned source studies behind evidence syntheses may include those that do not comply with responsible research conduct as genuine data.

**Figure 2:**
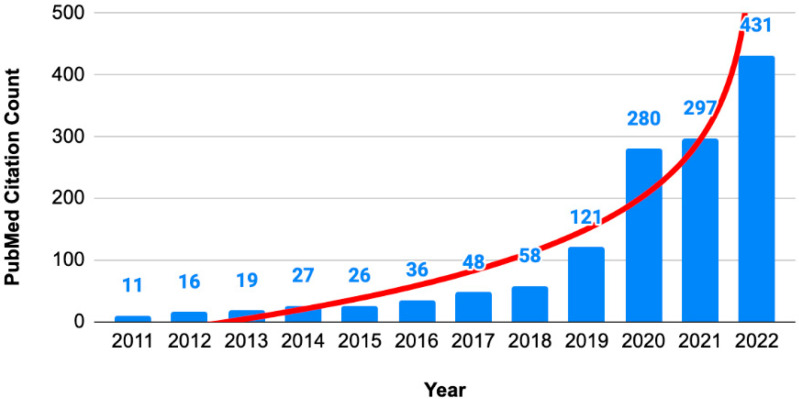
Numbers of articles with expressions of concern. Annual citation counts of expression of concern about articles in PubMed database.

The incorporation of RCT integrity assessment in evidence syntheses is an important consideration because the number of studies with expressions of concern has been rising exponentially (Figure 2).

**Figure 3:**
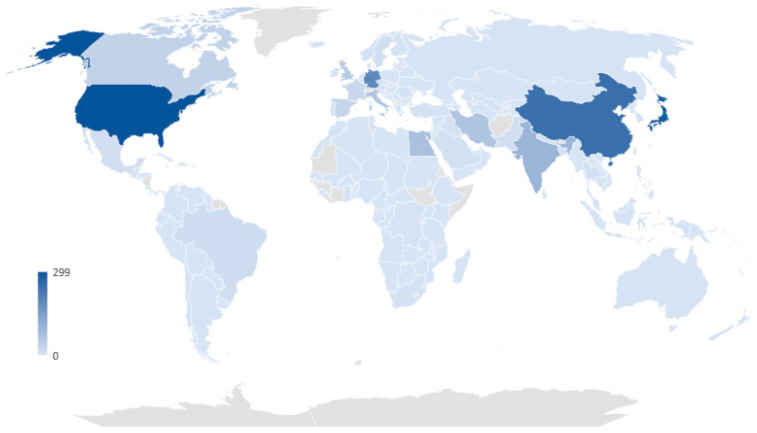
The number of retracted clinical studies per country based on Retraction Watch Database (http://retractiondatabase.org, data extracted on 2 February 2023).

Based on data retrieved on 2 February 2023, from the Retraction Watch Database, the top five countries with the highest number of retracted clinical studies per country are the United States, Japan, China, Germany, and India, with 299, 281, 245, 192, and 98 retractions, respectively. This data indicates that the retraction of studies is a global issue, and developed countries bear a great deal of responsibility for studies lacking integrity (Figure 3).

These issues, in turn, raise concerns about the integrity of evidence syntheses as inclusion of studies with retractions due to issues in “data/analyses/results” lead to summary estimates in systematic reviews that depart from the studies without these issues [25].

## 4. Importance of Assessing the Integrity of RCTs Included in Evidence Syntheses

In this paper, we focus on evidence syntheses that deploy RCTs, as it is a high-validity study design to underpin evidence-based medicine. We focus specifically on potential data-related integrity issues in RCTs. Behind every disease prevention and treatment breakthrough, there are thousands of volunteer participants in RCTs whose data are collated in evidence syntheses. Despite the need for obtaining ethics approval, confirming informed consent, and applying independent oversight during trial conduct, RCTs are not exempt from the possibility of (un)intentional integrity deviations. The general fact that expressions of concern (Figure 2) and retractions (Figure 3) are numerous has shaken public confidence, being markedly astounding during the COVID-19 pandemic [26]. It is likely that not all retractions are the result of deliberate fraud, falsification, and fabrication. Unintentional errors, spin, or flawed techniques are bound to have played their part [27,28]. However, every RCT with integrity concerns that remains usable poses a threat to patients and public health. Therefore, systematic reviewers and guideline developers need to be vigilant about problematic or “zombie” RCTs [29].

Evidence syntheses affected by inclusion of RCTs that have integrity deficits are not difficult to find. The need for change in the attitude towards integrity assessment within reviews is highlighted by the following examples. Recently, Hill et al. retracted a meta-analysis of RCTs concerning COVID-19. The significant benefits initially observed could not be sustained after several of the included studies in the meta-analysis were withdrawn due to fraudulent data or other additional problems [30,31]. Avenell et al. reviewed the impact of the inclusion of retracted RCTs on evidence syntheses [29]. This group of retracted RCTs were published in the late nineties, and the reason for the retractions was serious misconduct, including concerns related to data integrity. RCT retractions in this case were only applied nearly two decades after publication [32]. Following the retractions, at least one of the retracted RCTs had been included in 32 evidence syntheses published. Avenell et al. judged that the conclusion of 13/32 evidence syntheses would have changed if the retracted RCTs had been excluded. Marret et al. reviewed the impact of the inclusion of another group of retracted RCTs on evidence syntheses and judged that the conclusion of 2/14 would have changed if the retracted RCTs had been excluded [33]. Habib et al., in a commentary concerning the impact of the inclusion of yet another group of retracted RCTs on evidence syntheses, indicated that the likelihood of compromise was modest with some systematic reviews that performed sensitivity analyses, noting that their conclusions were different after excluding the retracted data [34]. Fanelli et al. similarly concluded that the potential epistemic cost of retraction was modest, with emphasis on the reason for retraction as the key issue [25].

These examples confirm that a specific methodology is required to address the issue of RCT integrity in systematic reviews head-on. It is important to recognize that the purpose of this methodology ought to focus on the protection of patients and public health. The precursors of failure to comply with responsible research conduct are many, including misconduct, recklessness, carelessness, lack of training, etc. [35]. It is not the systematic reviewers’ role to judge original authors’ motivations; journals, employers, funders, etc. have investigative and sanctioning roles. What systematic reviewers need is a proactive attitude towards synthesizing evidence that does not harbor integrity deviations [27]. This will protect the trustworthiness of evidence syntheses and evidence-based medicine.

## 5. How to Incorporate RCT Integrity Assessment in Evidence Syntheses

**Figure 4:**
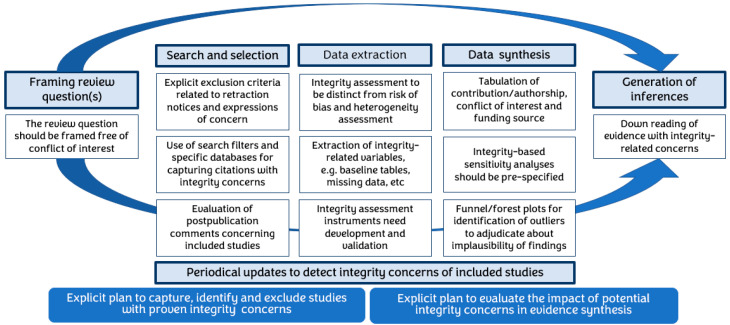
Infographic of the key steps during evidence synthesis to maximize the integrity of included research.

A systematic approach is required on various evidence synthesis fronts, including but not limited to the identification of honestly conducted research in searching, assessment of integrity (separately from bias assessments) of included studies, prior planning of sensitivity analyses for integrity, and transparency in generating inferences, given the cautious possibility of compromise in underlying data (Figure 4).

To assess the integrity of included RCTs, it is important to follow rigorous tool development methodology that has been widely applied previously in the development of study quality or risk of bias assessment tools [36,37]. Once developed, these should be fed back into revisions of current reporting guidelines [38]. Statistical analyses would need to be refocused, addressing integrity issues, e.g., funnel plot analyses may be used to inspect small studies that have implausibly large effects. In study-level published data meta-analyses, sub-group and meta-regression analyses may routinely include an integrity assessment-based variable. In individual patient data meta-analyses, statistical techniques can be applied for the detection of anomalous patterns in the underlying numerical data to check for data integrity [39,40,41,42]. These more sophisticated analyses should feed into evidence grading for the generation of judicious inferences.

## 6. Implications, Issues, Challenges, and Limitations

Evidence synthesis, mindful of research integrity, will need to attempt to collate all empirical evidence that fits pre-specified eligibility criteria, excluding studies with proven integrity concerns, using explicit methods to detect and quantify these concerns, evaluating their impact in planned statistical analyses and minimizing or eliminating the pollution of the inferences that may arise due to inclusion of studies with possibly compromised data. Periodical updates of reviews to detect integrity concerns of included studies should be performed (Table 2).

There are many issues to consider. A controversial aspect here is whether to include studies with expressed concerns but without proven misconduct. This controversy is not too different to the inclusion of RCTs with varying levels of risk of bias arising due to faulty randomization, lack of allocation concealment, or blinding. This is now routine in effectiveness reviews. The development, validation, and application of advanced methods that can accurately detect integrity breaches in publicly available RCTs are needed [43,44,45]. Handling the integrity assessment of selected studies after excluding those with confirmed integrity breaches requires further consideration in methodological development of evidence syntheses as, at present, there are no clear procedures established. We could only find one article providing a method for detecting retracted literature cited in systematic reviews and meta-analyses [46]. Unfortunately, there are no validated tools for integrity assessment yet [40]. One important aspect to highlight is that to date, there is no standard definition of the term “research integrity”. Thus, the concepts of bias, quality, validity, and integrity can be confusing for readers as well as reviewers. A precise characterization of research integrity distinct from the idea of risk of bias assessments is needed as a starting point for the required methodological developments to take place in the right direction in the future.

A particularly important issue that impinges on the critical appraisal of integrity is the enormous literature size and growth of publication rates. In 2015, there were about 28,100 and 6450 English-language and non-English-language science, technology, and medicine journals, respectively, growing at about 3% annually [47]. Defective studies should never get to enter circulation, but the growing volume complicates the challenge. To put a lid on the integrity-related concern that will grow with this literature expansion, automated checks will be required just as they have been used for the detection of plagiarism. This is required in part because the peer review process might not be able to cope with new ways of capturing defective literature since editors and peer reviewers would have to upgrade their knowledge/skill sets [7]. Computer sciences are being deployed for critical appraisal [48].

## 7. Artificial Intelligence for Integrity Assessment

The inclusion of efficient tools that will automate integrity assessment in evidence synthesis is the next methodological advance required. Review projects usually require a team of reviewers who screen and identify literature and evaluate included study quality. They will additionally need to perform integrity assessments before collating findings and generating recommendations. Currently, reviews take up human effort and take too long to collect and evaluate the data included, undertaking double-checks to minimize errors. The review process is estimated to take, on average, 67.3 weeks (IQR 41.6) to complete an evidence synthesis, and publishing the synthesis involves 5.3 review team members (IQR 3), on average [49]. In addition to being slow, there is an inherent error rate associated with human effort, e.g., the selection process suffers a 10% error (false inclusion and false exclusion) rate [50]. Thus, it has been concluded that: “*Systematic reviews presently take much time and require large amounts of human resources. In the light of the ever-increasing volume of published studies, application of existing computing and informatics technology should be applied to decrease this time and resource burden*” [49]. The use of automation is also emphasized for integrity assessment [27] and, although infrequently used until now, it is impactful [51].

These assessments need development and validation of new instruments to enable the detection and exclusion of questionable evidence from evidence syntheses, without the need to wait for retractions. Automated detection of retractions, specifically for data-related misconduct associated with fabrication, falsification, and other types of forgery, are needed [44]. This way, integrity assessments in systematic reviews will streamline the literature correction process and may include alerts for journals to trigger investigations. The tools, once developed and validated, may also be used for improving peer-reviews, reducing the circulation of “zombie” trials. This will improve the validity of the evidence syntheses going forward and will assist in the pre- and post-publication review process in cases of allegations.

## 8. Current Conclusions

Evidence syntheses collating RCTs influence practice and health policies, directly impacting patient care. The investigation and retraction of RCTs with integrity concerns is a slow process. Thus, defective RCTs remain in circulation, putting patients at risk. Even after retraction, defective studies continue to be cited in systematic reviews as they are not removed from databases and their signposting is poor. Evidence syntheses fail to issue corrections even when retractions are identified. All this entails risk as patients remain exposed to interventions that are futile or even risky for their health. Evidence syntheses need to urgently upgrade their methods to incorporate integrity assessments as a routine, as outlined in Figure 4.

## 9. Future Direction

Research integrity, a broad concept holistically incorporating both ethical and professional standards [18], needs to be considered in evidence synthesis covering the whole range of issues inherent in responsible RCT conduct. This needs to be defined explicitly through further research. However, the illegal Tuskegee syphilis experiment from the recent past is a case in point where unethical research can permeate within the literature without comment. In a 2022 journal article [51], Tobin wrote: “*Despite 15 journal articles detailing the results, no physician published a letter criticizing the Tuskegee study. Informed consent was never sought; instead, Public Health Service researchers deceived the men into believing they were receiving expert medical care*”. These articles remain formally unretracted from the literature to this date. Note that ethics and consent standards were not covered by us in this article as we focused on data-related integrity, but it remains a key aspect demanding future research and development for its proper implementation and monitoring in RCTs. There are many articles showing deficits in informed consents in clinical studies [52,53,54], and this type of integrity assessment ought to be featured in evidence syntheses.

There ought to be an emphasis on prevention, metaphorically nipping the evil studies in the bud [55]. This begins with clarifying integrity-related definitions, e.g., what are questionable research practices that raise integrity concerns. Then, it would be appropriate to identify modifiable factors and barriers that may affect best practice compliance [3]. Future direction would take the above forward emphasizing routine adherence to ethical and professional standards through periodic integrity-specific training tailored to educational environment for all stakeholders involved in the RCT research lifecycle, including but not limited to researchers, ethics committee members, funders, editors, peer reviewers, systematic reviewers, guideline makers, drug regulators, medical journalists, as well as lay readers. Integrity training in clinical trials has been recommended in a recent international multi-stakeholder consensus statement [44], with emphasis on enabling research teams from low resource settings to make contributions. The creation of solid systems backed by valid and robust instruments and methods for inculcating research integrity are urgently needed. Future evidence syntheses will directly benefit from these improvements in the integrity of the conduct, analysis, and reporting of primary RCTs collated within literature reviews.

## Figures and Tables

**Table 1:** Research integrity related terms and definitions.

Research Integrity Terms	Definition of Terms *
Research integrity	Undertaking research in accordance with ethical and professional principles and standards.
Integrity principles	A set of values and concepts for guiding researcher behavior.
Integrity standards	Specifications of conduct that must be adhered to when participating in or carrying out research.
Bias	Systematic error that invalidates the observed effects in trials due to flaws in methodological aspects such as failure to concealment of randomization, lack of blinding, etc. Bias is distinct from data-related integrity flaws that arise due to misconduct.
Moral Values	The set of principles and standards that differentiate “right” from “wrong”.
Bioethics	Making choices in biomedical research around what are “right” and “wrong” values and behaviors.
Anti-whistleblower	Individuals who do not report nor prevent another individual from reporting known research misconduct.
Dishonesty	Behaviors that transgress moral values and bioethical standards
Duplication	A redundant publication that recycles or borrows content from authors own previous work without citation (see also self-plagiarism).
Ethics	Decision making based on moral and bioethical principles to protect those subjects of research and wider society.
Ethical Misdemeanors	Unacceptable or improper behavior that violates formal regulations.
Expression of concern	Note issued by journal editors or publishers to make readers aware that there is a concern about the integrity of a particular published article.
Fabrication, Falsification, Plagiarism (FFP)	The unholy trinity of misconduct in education, research or scholarship. Plagiarism is not the focus of this paper.
Fabrication	Making up data, experiments, or other significant information in proposing, conducting, or reporting research and using them as if genuine.
Falsification	Forging research content, images, data, equipment, or processes in the way that they are inaccurately represented.
Forgery	Forging content, images, data, equipment, or processes in the way that they are inaccurately represented.
Fraud	Any intentional act of deception in research violating research ethics.
Infringement	Breach of good practice occurring from questionable, unlawful or unethical behavior.
Irresponsible Research Practices	Practices that are regarded as unethical but fall short of being considered research misconduct
Masking	Subset of data falsification consisting of minimizing or omitting data which does not support desired conclusions or results.
Misconduct	Unethical or unprofessional behavior in research.
Negligence	Failure to follow the required standard that results in harm to a person or organization.
Plagiarism	Presenting the work of others as if it were own work without proper acknowledgment or citation of the original source. Plagiarism is not the focus of this paper.
Self-plagiarism	Auto-plagiarism, i.e., the author adds insignificant additional data or information to previously published work changing title, modifying aim of the study or recalculating results, with the omission of citation to own previous publications. Self-plagiarism is not the focus of this paper.
Questionable research practices (QRPs)	Research practices that are unethical but fall short of being considered research misconduct.
Recycle	Recycling or borrowing content from authors own previous work without citation.
Redundant Publication	A published work (or substantial sections from a published work) is/are published more than once (in the same or another language) without adequate acknowledgment of the source/cross-referencing/justification. It is also when the same (or substantially overlapping) data is presented in more than one publication without adequate cross-referencing/justification, particularly when this is done in such a way that reviewers/readers are unlikely to realise that most or all the findings have been published before.
Replication	Repeating a piece of research in order to verify and/or complement the original results.
Retraction	Withdrawing or removing a published paper from the research record because of a variety of reasons including a post-publication reassessment showing that the data or results reported are unreliable or because the paper involves research misconduct. Journals publish retraction notices and identify retracted papers in electronic databases with reasons for retraction no always clearly stated.
Transgression	Breach of good practice occurring from questionable, unlawful or unethical behavior.
Transparency	Openness about activities and related decisions that affect academia and society and willingness to communicate these in a clear, accurate, timely, honest and complete manner.
Violation/Breach	Breach of responsible research practices due to questionable, unlawful or unethical behavior in the conduct, analysis and reporting of research.
Conflict of interest	Potential or perceived compromise in judgement or objectivity due to financial or personal relationships or other considerations.
Confidentiality Violation	Disclosing to others information received in confidence without prior, explicit authorization of the person to whom the information belongs.
Author’s Ethical Rights	The right to vindicate the ownership of work and assure its integrity and genuine status
Authorship Abuse	Any kind of authorship attribution not based on genuine contribution.
Authorship Coercion	An authorship that is demanded rather than voluntarily awarded.
Ghost Authorship	The practice of using a non-named (merited, but not listed) author to write or prepare a text for publication.
Invented Authorship	Naming a fictitious person, a colleague or a stranger as a co-author without permission.
Unethical Authorship	Crediting a person who has not contributed to the research in authorship; excluding from authorship a genuine contributor; manipulating the sequence of authors in an unjustified and improper way; removing names of contributors in subsequent publications; using one’s power to insist on being added as an author without any contribution; including an author without their permission.

* Adapted from “Glossary for Research and Academic Ethics and Integrity (https://h2020integrity.eu/glossary), accessed on 26 February 2018 [19], “Errata, retractions, partial retractions, corrected and republished articles, duplicate publications, comments (including author replies), updates, patient summaries, and republished (reprinted) articles policy for MEDLINE (fact sheet)” 2015 (https://www.nlm.nih.gov/pubs/factsheets/errata.html), accessed on 26 February 2018 [20]. COPE Forum: Expressions of concern (https://publicationethics.org/resources/forum-discussions/expressions-of-concern), accessed on 26 February 2018 [21] and Khan, K.S.; Zamora, J. Systematic Reviews to Support Evidence-Based Medicine, 3rd ed.; Taylor & Francis Publishing: London, UK, 2022 [1].

**Table 2:** Steps of evidence synthesis and key integrity related issues.

Steps of Evidence Synthesis	Integrity Related Issues
Framing question	Review question should be framed free of conflict of interest and should specify its focus on including studies with integrity
Search and selection	Explicit exclusion criteria related to retraction notices and expressions of concern about integrity should be pre-specified
	Specific retraction and integrity concern searches should be deployed, e.g., in Retraction Watch Database
	Search filters for capturing citations with integrity concerns should be developed and used
	Evaluation of post-publication comments concerning included studies should be sought and evaluated, e.g., letters to editors, Pub-Peer comments, etc.
Data extraction	Specific data extraction to permit integrity assessment, e.g., baseline tables, missing data, etc.
	Integrity assessment needs to be distinct from risk of bias and heterogeneity assessment
	Integrity assessment instruments need development and validation
	Developed integrity assessment instruments need automation
Data synthesis	Tabulation of contribution/authorship, conflict of interest and funding source, etc. related to integrity should be routine
	Integrity-based sensitivity analyses should be pre-specified
	Use of funnel plots to look for outliers should additionally be pre-specified with delineation of threshold for defining implausibly extreme results
Inference generation	Down grading of evidence with integrity concerns should be explicitly deployed in generation of recommendations
Updates	Periodical updates of reviews to detect integrity concerns of included studies and issuing correction notices

## Data Availability

Not applicable.

## References

[1] Khan K.S., Zamora J. (2022). Systematic Reviews to Support Evidence-Based Medicine.

[2] De Vrieze J. (2021). Large survey finds questionable research practices are common. Science.

[3] Gopalakrishna G., ter Riet G., Vink G., Stoop I., Wicherts J.M., Bouter L.M. (2022). Prevalence of questionable research practices, research misconduct and their potential explanatory factors: A survey among academic researchers in the Netherlands. PLoS ONE.

[4] Steen R.G., Casadevall A., Fang F.C. (2013). Why Has the Number of Scientific Retractions Increased?. PLoS ONE.

[5] Vinkers C.H., Lamberink H.J., Tijdink J.K., Heus P., Bouter L., Glasziou P., Moher D., Damen J.A., Hooft L.O.W. (2021). The methodological quality of 176,620 randomized controlled trials published between 1966 and 2018 reveals a positive trend but also an urgent need for improvement. PLoS Biol..

[6] Ioannidis J.P.A. (2021). Hundreds of thousands of zombie randomised trials circulate among us. Anaesthesia.

[7] Teixeira da Silva J.A. (2022). A Synthesis of the Formats for Correcting Erroneous and Fraudulent Academic Literature, and Associated Challenges. J. Gen. Philos. Sci..

[8] Higgins G. (2011). Cochrane Handbook for Systematic Reviews of Interventions.

[9] Twells L.K. (2021). Evidence-Based Decision-Making 1: Critical Appraisal. Methods Mol. Biol..

[10] World Medical Association (WMA) Declaration on Guidelines for Continuous Quality Improvement in Healthcare. https://www.wma.net/policies-post/wma-declaration-on-guidelines-for-continuous-quality-improvement-in-health-care/.

[11] ICH Official Web Site: ICH. https://www.ich.org/.

[12] Bauchner H., Golub R.M., Fontanarosa P.B. (2019). Reporting and Interpretation of Randomized Clinical Trials. JAMA.

[13] Stavale R., Ferreira G.I., Galvão J.A.M., Zicker F., Novaes M.R.C.G., De Oliveira C., Guilhem D. (2019). Research misconduct in health and life sciences research: A systematic review of retracted literature from Brazilian institutions. PLoS ONE.

[14] Shea B.J., Reeves B.C., Wells G., Thuku M., Hamel C., Moran J., Moher D., Tugwell P., Welch V., Kristjansson E. (2017). AMSTAR 2: A critical appraisal tool for systematic reviews that include randomised or non-randomised studies of healthcare interventions, or both. BMJ.

[15] Whiting P., Savović J., Higgins J.P., Caldwell D.M., Reeves B.C., Shea B., Davies P., Kleijnen J., Churchill R., ROBIS group (2016). ROBIS: A new tool to assess risk of bias in systematic reviews was developed. J. Clin. Epidemiol..

[16] Molino C.d.G.R.C., Ribeiro E., Romano-Lieber N.S., Stein A.T., de Melo D.O. (2017). Methodological quality and transparency of clinical practice guidelines for the pharmacological treatment of non-communicable diseases using the AGREE II instrument: A systematic review protocol. Syst. Rev..

[17] Chen Y., Yang K., Marušić A., Qaseem A., Meerpohl J.J., Flottorp S., Akl E.A., Schünemann H.J., Chan E.S.Y., Falck-Ytter Y. (2017). A reporting tool for practice guidelines in health care: The RIGHT statement. Ann. Intern. Med..

[18] Steneck N.H. (2006). Fostering integrity in research: Definitions, current knowledge, and future directions. Sci. Eng. Ethics.

[19] H2020 INTEGRITY—Glossary. https://h2020integrity.eu/resources/glossary/.

[20] National Library of Medicine (2015). Errata, Retractions, and Other Linked Citations in PubMed. http://wayback.archive-it.org/org-350/20180312141525/https://www.nlm.nih.gov/pubs/factsheets/errata.html.

[21] COPE COPE Forum 26 February 2018: Expressions of Concern. https://publicationethics.org/resources/forum-discussions/expressions-of-concern.

[22] Malički M., Jerončić A., Aalbersberg I.J.J., Bouter L., ter Riet G. (2021). Systematic review and meta-analyses of studies analysing instructions to authors from 1987 to 2017. Nat Commun..

[23] Schneider J., Ye D., Hill A.M., Whitehorn A.S. (2020). Continued post-retraction citation of a fraudulent clinical trial report, 11 years after it was retracted for falsifying data. Scientometrics.

[24] Kataoka Y., Banno M., Tsujimoto Y., Ariie T., Taito S., Suzuki T., Oide S., Furukawa T.A. (2022). Retracted randomized controlled trials were cited and not corrected in systematic reviews and clinical practice guidelines. J. Clin. Epidemiol..

[25] Fanelli D., Wong J., Moher D. (2022). What difference might retractions make? An estimate of the potential epistemic cost of retractions on meta-analyses. Account. Res..

[26] Fleming T.R., Labriola D., Wittes J. (2020). Conducting Clinical Research During the COVID-19 Pandemic: Protecting Scientific Integrity. JAMA.

[27] Núñez-Núñez M., Andrews J.C., Fawzy M., Bueno-Cavanillas A., Khan K.S. (2022). Research integrity in clinical trials: Innocent errors and spin versus scientific misconduct. Curr. Opin. Obstet. Gynecol..

[28] Fletcher R.H.B.B. (2007). “Spin” in scientific writing: Scientific mischief and legal jeopardy. Med. Law.

[29] Avenell A., Stewart F., Grey A., Gamble G., Bolland M. (2019). An investigation into the impact and implications of published papers from retracted research: Systematic search of affected literature. BMJ Open.

[30] Hill A., Mirchandani M., Pilkington V. (2022). Ivermectin for COVID-19: Addressing Potential Bias and Medical Fraud. Open Forum Infect. Dis..

[31] Hill A., Garratt A., Levi J., Falconer J., Ellis L., McCann K., Pilkington V., Qavi A., Wang J., Wentzel H. (2021). Retracted: Meta-analysis of Randomized Trials of Ivermectin to Treat SARS-CoV-2 Infection. Open Forum Infect. Dis..

[32] Bolland M.J., Avenell A., Gamble G.D., Grey A. (2016). Systematic review and statistical analysis of the integrity of 33 randomized controlled trials. Neurology.

[33] Marret E., Elia N., Dahl J.B., McQuay H.J., Møiniche S., Moore R.A., Straube S., Tramèr M.R. (2009). Susceptibility to fraud in systematic reviews: Lessons from the reuben case. Anesthesiology.

[34] Habib A.S.G.T. (2013). Scientific fraud: Impact of Fujii’s data on our current knowledge and practice for the management of postoperative nausea and vomiting. Anesth. Analg..

[35] Resnik D.B., Smith E.M., Chen S.H.G.C. (2017). What is Recklessness in Scientific Research? The Frank Sauer Case. Account. Res..

[36] Sterne J.A.C., Savović J., Page M.J., Elbers R.G., Blencowe N.S., Boutron I., Cates C.J., Cheng H.Y., Corbett M.S., Eldridge S.M. (2019). RoB 2: A revised tool for assessing risk of bias in randomised trials. BMJ.

[37] Jüni P., Witschi A., Bloch R., Egger M. (1999). The hazards of scoring the quality of clinical trials for meta-analysis. JAMA.

[38] Schulz K.F., Altman D.G., Moher D. (2010). CONSORT 2010 statement: Updated guidelines for reporting parallel group randomized trials. Ann. Intern. Med..

[39] van den Bor R.M., Vaessen P.W.J., Oosterman B.J., Zuithoff N.P.A., Grobbee D.E., Roes K.C.B. (2017). A computationally simple central monitoring procedure, effectively applied to empirical trial data with known fraud. J. Clin. Epidemiol..

[40] Pogue J.M., Devereaux P.J., Thorlund K., Yusuf S. (2013). Central statistical monitoring: Detecting fraud in clinical trials. Clin. Trials.

[41] de Viron S., Trotta L., Schumacher H., Hans-Juergen L., Höppner S., Young S., Buyse M. (2022). Detection of Fraud in a Clinical Trial Using Unsupervised Statistical Monitoring. Ther. Innov. Regul. Sci..

[42] O’Kelly M. (2004). Using statistical techniques to detect fraud: A test case. Pharm. Stat..

[43] Núñez-Núñez M., Maes-Carballo M., Mignini L.E., Chien P.F.W., Khalaf Y., Fawzy M., Zamora J., Khan K.S., Bueno-Cavanillas A. (2023). Research integrity in randomized clinical trials: A scoping umbrella review. Int. J. Gynecol. Obstet..

[44] Khan K.S. (2023). Cairo Consensus Group on Research Integrity. International multi-stakeholder consensus statement on clinical trial integrity. BJOG.

[45] Khan K.S., Fawzy M., Chien P.F.W. (2023). Integrity of randomized clinical trials: Performance of integrity tests and checklists requires assessment. Int. J. Gynaecol. Obstet..

[46] Morán J.M., Santillán-García A., Herrera-Peco I. (2022). SCRUTATIOm: How to detect retracted literature included in systematics reviews and metaanalysis using SCOPUS© and ZOTERO©. Gac. Sanit..

[47] Ware M., Mabe M. (2015). The STM Report: An Overview of Scientific and Scholarly Journal Publishing Fourth Edition.

[48] The Systematic Review Toolbox. http://systematicreviewtools.com/software.php.

[49] Borah R., Brown A.W., Capers P.L., Kaiser K.A. (2017). Analysis of the time and workers needed to conduct systematic reviews of medical interventions using data from the PROSPERO registry. BMJ Open.

[50] Wang Z., Nayfeh T., Tetzlaff J., O’Blenis P., Murad M.H. (2020). Error rates of human reviewers during abstract screening in systematic reviews. PLoS ONE.

[51] Tercero-Hidalgo J.R., Khan K.S., Bueno-Cavanillas A., Fernández-López R., Huete J.F., Amezcua-Prieto C., Zamora J., Fernández-Luna J.M. (2022). Artificial intelligence in COVID-19 evidence syntheses was underutilized, but impactful: A methodological study. J. Clin. Epidemiol..

[52] Pietrzykowski T., Smilowska K. (2021). The reality of informed consent: Empirical studies on patient comprehension—Systematic review. Trials.

[53] Schellings R., Kessels A.G., ter Riet G., Knottnerus J.A., Sturmans F. (2006). Randomized consent designs in randomized controlled trials: Systematic literature search. Contemp. Clin. Trials.

[54] Timmermann C., Orzechowski M., Kosenko O., Woniak K., Steger F. (2022). Informed Consent in Clinical Studies Involving Human Participants: Ethical Insights of Medical Researchers in Germany and Poland. Front. Med..

[55] Khan K.S. (2021). Comment on Khan: “Flawed Use of Post Publication Data Fabrication Tests’. Research Misconduct Tests: Putting Patients” Interests First. J. Clin. Epidemiol..

